# Supplementary material for: Parabacteroides distasonis ameliorates insulin resistance via activation of intestinal GPR109a
Source: Nat Commun. 2023 Nov 25;14:7740. doi: 10.1038/s41467-023-43622-3 (PMC10676405; doi:10.1038/s41467-023-43622-3)
Supplement: Supplementary file 1 — Supplementary Information [file 41467_2023_43622_MOESM1_ESM.pdf]

Parabacteroides distasonis ameliorates insulin resistance via activation of intestinal GPR109a

Yonggan Sun[1, 2, 3, &], Qixing Nie[1, 2, 3, &], Shanshan Zhang[1, 2, 3], Huijun He[1, 2, 3], Sheng Zuo[1, 2, 3], Chunhua Chen[1, 2, 3], Jingrui Yang[1, 2, 3], Haihong Chen[1, 2, 3], Jielun Hu[1, 2, 3], Song Li[1, 2, 3], Jiaobo Cheng[1], Baojie Zhang[1], Zhitian Zheng[1], Shijie Pan[1], Ping Huang[4], Lu Lian[4], Shaoping Nie[1, 2, 3 *]

[1] State Key Laboratory of Food Science and Resources, Nanchang University, Nanchang, China

[2] China-Canada Joint Lab of Food Science and Technology, Nanchang University, Nanchang, China

[3] Key Laboratory of Bioactive Polysaccharides of Jiangxi Province, Nanchang University, Nanchang, China

[4] Department of Nutrition, the First Affiliated Hospital of Nanchang University, Nanchang, China

[&] These authors contributed equally: Yonggan Sun, Qixing Nie

[*] Correspondence: spnie@ncu.edu.cn

Supplemental Figures and Legends

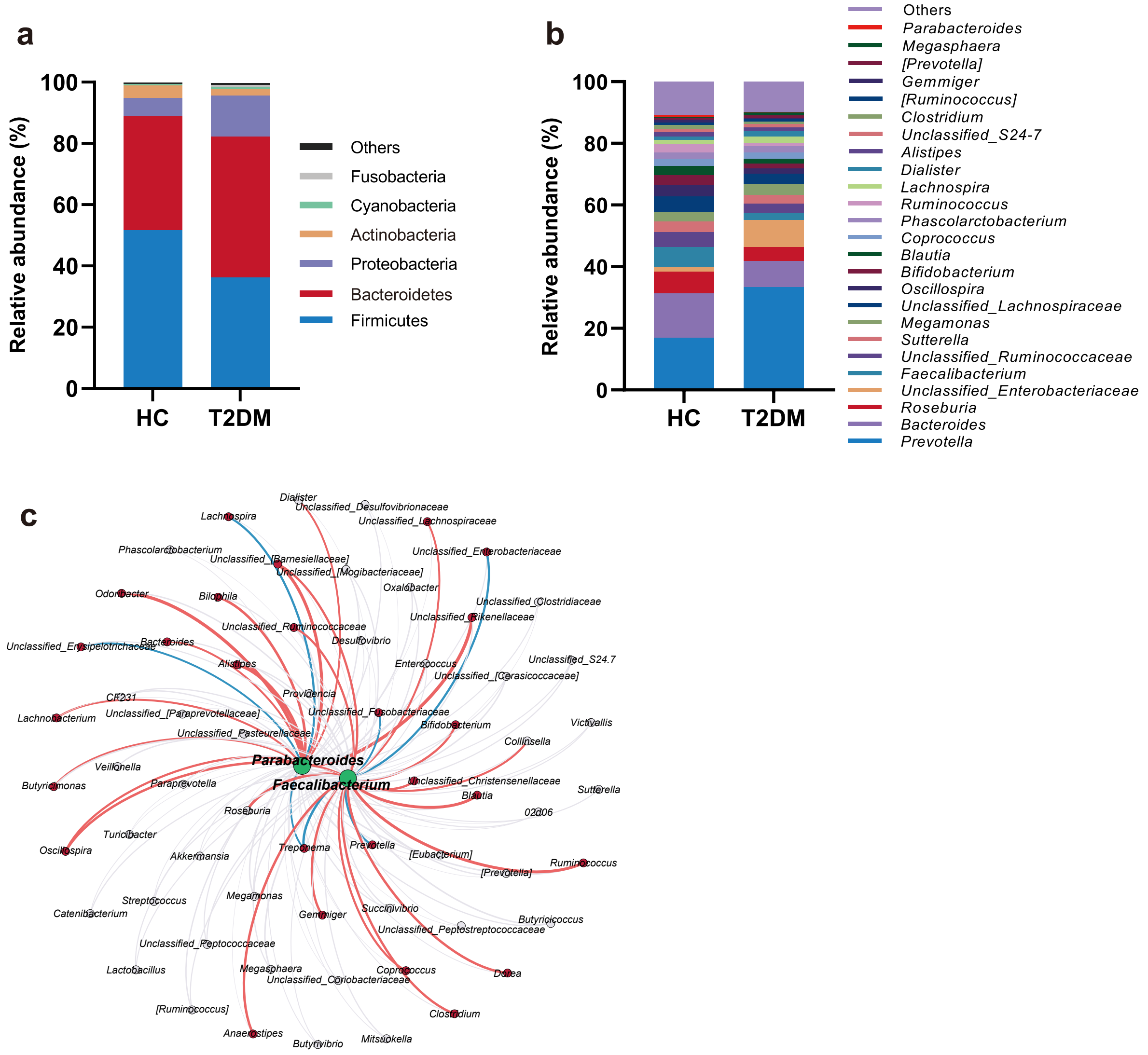

Supplementary Fig. 1 Gut microbiota profiling in patients with T2DM

a-b Relative abundance of bacteria at the phylum (**a**) and genus (**b**) level. **c** Gut microbial co-occurrence network analysis indicated *Parabacteroides* and *Faecalibacterium* are core genus (average relative abundance > 0.1%) in gut microbiota of humans (T2DM and HC groups). The red line indicates Spearman's rank (two-tailed Spearman's rank test) correlation coefficient > 0.30 and *P* < 0.05; the blue line indicates Spearman's rank correlation coefficient < -0.30 and *P* < 0.05. T2DM (type 2 diabetes mellitus); HC (health control). Source data can be found in Source Data file.

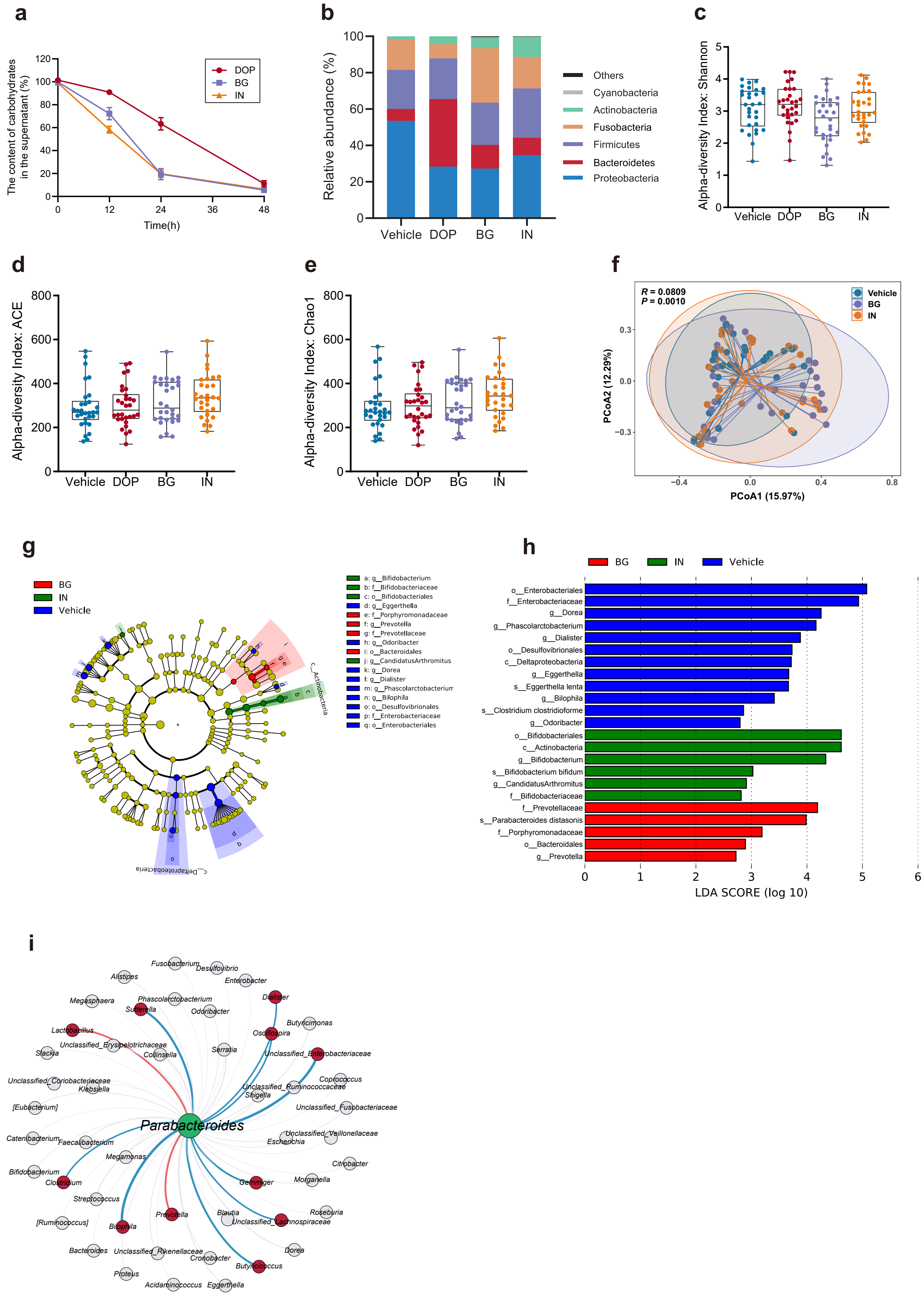

a
The content of carbohydrates in the supernatant (%)
Time(h)
DOP
BG
IN
b
Relative abundance (%)
Vehicle
DOP
BG
IN
Others
Cyanobacteria
Actinobacteria
Fusobacteria
Firmicutes
Bacteroidetes
Proteobacteria
c
Alpha-diversity Index: Shannon
d
Alpha-diversity Index: ACE
e
Alpha-diversity Index: Chao1
f
R = 0.0809
P = 0.0010
PCoA1 (15.97%)
PCoA2 (12.29%)
g
c__Actinobacteria
c__Deltaproteobacteria
a: g__Bifidobacterium
b: f__Bifidobacteriaceae
c: o__Bifidobacteriales
d: g__Eggerthella
e: f__Porphyromonadaceae
f: g__Prevotella
g: f__Prevotellaceae
h: g__Odoribacter
i: o__Bacteroidales
j: g__CandidatusArthromitus
k: g__Dorea
l: g__Dialister
m: g__Phascolarctobacterium
n: g__Bilophila
o: o__Desulfovibrionales
p: f__Enterobacteriaceae
q: o__Enterobacteriales
h
o__Enterobacteriales
f__Enterobacteriaceae
g__Dorea
g__Phascolarctobacterium
g__Dialister
o__Desulfovibrionales
c__Deltaproteobacteria
g__Eggerthella
s__Eggerthella lenta
g__Bilophila
s__Clostridium clostridioforme
g__Odoribacter
o__Bifidobacteriales
c__Actinobacteria
g__Bifidobacterium
s__Bifidobacterium bifidum
g__CandidatusArthromitus
f__Bifidobacteriaceae
f__Prevotellaceae
s__Parabacteroides distasonis
f__Porphyromonadaceae
o__Bacteroidales
g__Prevotella
LDA SCORE (log 10)
i
Parabacteroides
Fusobacterium
Desulfovibrio
Enterobacter
Alistipes
Megasphaera
Phascolarctobacterium
Sutterella
Odoribacter
Dialister
Butyricimonas
Lactobacillus
Oscillospira
Unclassified_Erysipelotrichaceae
Slackia
Collinsella
Serratia
Unclassified_Enterobacteriaceae
Unclassified_Coriobacteriaceae
Klebsiella
Coprococcus
Unclassified_Ruminococcaceae
Shigella
[Eubacterium]
Unclassified_Fusobacteriaceae
Catenibacterium
Faecalibacterium
Unclassified_Veillonellaceae
Escherichia
Megamonas
Citrobacter
Bifidobacterium
Clostridium
Gemmiger
Morganella
Streptococcus
[Ruminococcus]
Prevotella
Blautia
Roseburia
Bilophila
Unclassified_Lachnospiraceae
Bacteroides
Unclassified_Rikenellaceae
Cronobacter
Dorea
Proteus
Butyricicoccus
Acidaminococcus
Eggerthella

Supplementary Fig. 2 Effect of DOP, IN, and BG on the composition of gut microbiota

a The changes of total carbohydrates content of medium (DOP, IN, and BG as the sole carbon) during *in vitro* fermentation with fecal microbiota from T2DM patients. n = 30 per group. **b** Relative abundance of bacteria at the phylum level in different groups. **c-e** α-diversity indexes in different groups as indicated by the Shannon, ACE, and Chao1 indexes. The box plots showing the minima, maxima, centre, bounds of box and whiskers and percentile. n = 30 per group. **f** Principal coordinate analysis (PCoA) of the bacteria in different groups by weighted UniFrac distance (ANOSIM test). n = 30 per group. **g** Taxonomic cladogram generated from LEfSe analysis. Each circle's size is proportional to the taxon's abundance. **h** LDA score represents the taxonomic data with significant differences between IN, BG, and Vehicle groups. Only LDA scores > 2 are shown. Green indicates enriched taxa in the IN group; red indicates enriched taxa in the BG group; blue indicates enriched taxa in the Vehicle group. **i** Gut microbial co-occurrence network analysis indicates *Parabacteroides* is core genus (average relative abundance > 0.1%) in gut microbiota after DOP fermentation. The red line indicates Spearman's rank (two-tailed Spearman's rank test) correlation coefficient > 0.30 and $P < 0.05$; the blue line indicates Spearman's rank correlation coefficient < -0.30 and $P < 0.05$. DOP (*Dendrobium officinale* polysaccharide); BG (β-glucan); IN (inulin). Source data can be found in Source Data file.

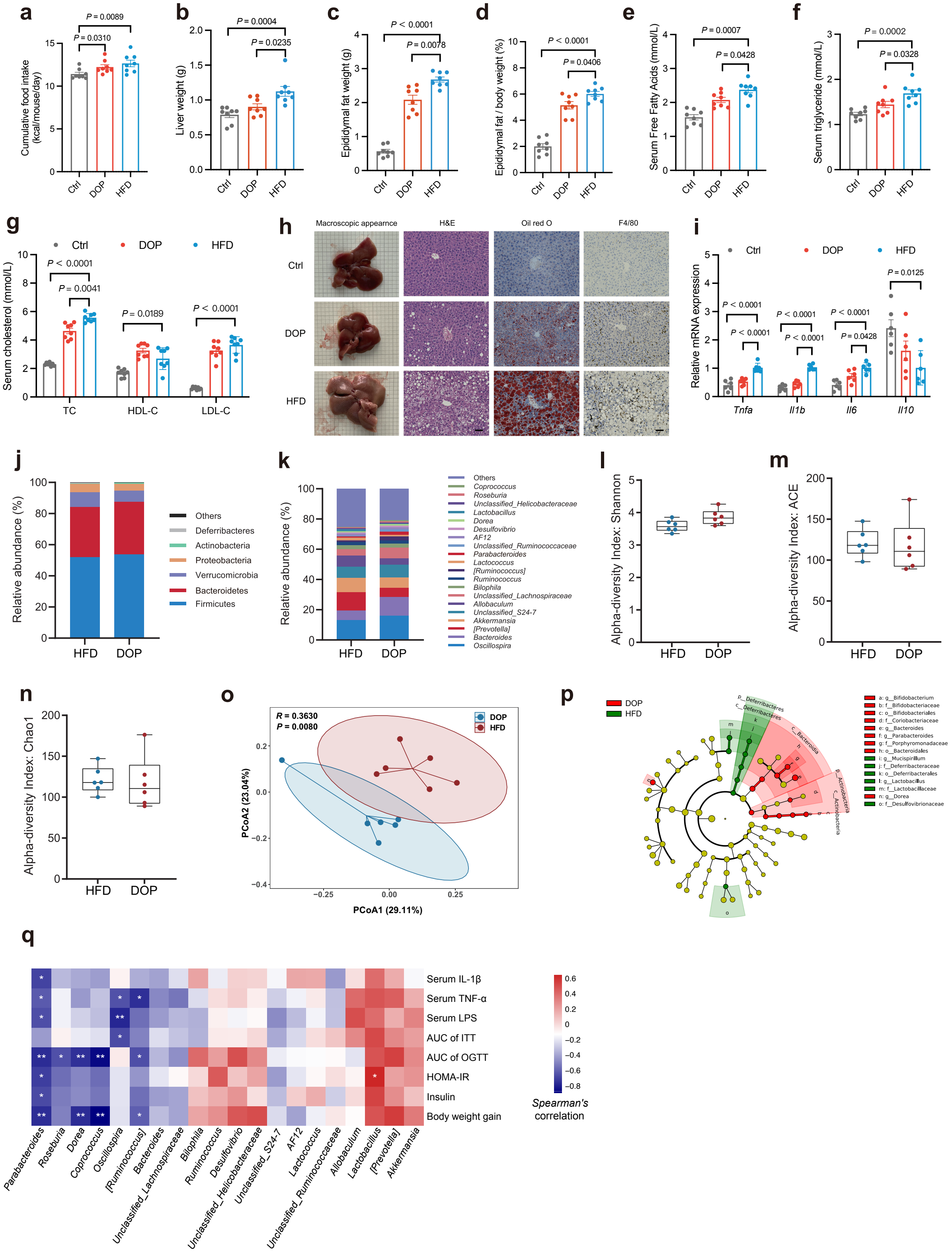

a
P = 0.0089
P = 0.0310
Cumulative food intake (kcal/mouse/day)
Ctrl
DOP
HFD
b
P = 0.0004
P = 0.0235
Liver weight (g)
c
P < 0.0001
P = 0.0078
Epididymal fat weight (g)
d
P < 0.0001
P = 0.0406
Epididymal fat / body weight (%)
e
P = 0.0007
P = 0.0428
Serum Free Fatty Acids (mmol/L)
f
P = 0.0002
P = 0.0328
Serum triglyceride (mmol/L)
g
Ctrl
DOP
HFD
P < 0.0001
P = 0.0041
P = 0.0189
P < 0.0001
Serum cholesterol (mmol/L)
TC
HDL-C
LDL-C
h
Macroscopic appearnce
H&E
Oil red O
F4/80
Ctrl
DOP
HFD
i
Ctrl
DOP
HFD
P = 0.0125
P < 0.0001
P < 0.0001
P < 0.0001
P < 0.0001
P < 0.0001
P = 0.0428
Relative mRNA expression
Tnfa
Il1b
Il6
Il10
j
Relative abundance (%)
Others
Deferribacteres
Actinobacteria
Proteobacteria
Verrucomicrobia
Bacteroidetes
Firmicutes
HFD
DOP
k
Relative abundance (%)
Others
Coprococcus
Roseburia
Unclassified_Helicobacteraceae
Lactobacillus
Dorea
Desulfovibrio
AF12
Unclassified_Ruminococcaceae
Parabacteroides
Lactococcus
[Ruminococcus]
Ruminococcus
Bilophila
Unclassified_Lachnospiraceae
Allobaculum
Unclassified_S24-7
Akkermansia
[Prevotella]
Bacteroides
Oscillospira
HFD
DOP
l
Alpha-diversity Index: Shannon
HFD
DOP
m
Alpha-diversity Index: ACE
HFD
DOP
n
Alpha-diversity Index: Chao1
HFD
DOP
o
R = 0.3630
P = 0.0080
DOP
HFD
PCoA2 (23.04%)
PCoA1 (29.11%)
p
DOP
HFD
a: g__Bifidobacterium
b: f__Bifidobacteriaceae
c: o__Bifidobacteriales
d: f__Coriobacteriaceae
e: g__Bacteroides
f: g__Parabacteroides
g: f__Porphyromonadaceae
h: o__Bacteroidales
i: g__Mucispirillum
j: f__Deferribacteraceae
k: o__Deferribacterales
l: g__Lactobacillus
m: f__Lactobacillaceae
n: g__Dorea
o: f__Desulfovibrionaceae
p__Deferribacteres
c__Deferribacteres
c__Bacteroidia
p__Actinobacteria
c__Actinobacteria
q
Serum IL-1β
Serum TNF-α
Serum LPS
AUC of ITT
AUC of OGTT
HOMA-IR
Insulin
Body weight gain
Parabacteroides
Roseburia
Dorea
Coprococcus
Oscillospira
[Ruminococcus]
Bacteroides
Unclassified_Lachnospiraceae
Bilophila
Ruminococcus
Desulfovibrio
Unclassified_Helicobacteraceae
Unclassified_S24-7
AF12
Lactococcus
Unclassified_Ruminococcaceae
Allobaculum
Lactobacillus
[Prevotella]
Akkermansia
Spearman's correlation

Supplementary Fig. 3 Effects of DOP on HFD-induced IR mice

After an 8-week HFD treatment, mice were given PBS (HFD group) or DOP (DOP group) for 5 weeks, and the control group (Ctrl) was fed with a chow diet and given an equivalent volume of PBS. (a-g) n = 8 mice per group, (h-o) n = 6 mice per group. **a** Food intake. **b** Liver weight. **c** Epididymal fat weight. **d** Epididymal fat/body weight (%). **e** Serum free fatty acids. **f** Serum triglyceride. **g** Serum cholesterol. **h** Representative images of the appearance of liver, liver sections after H&E staining, oil red O staining, and F4/80 immunohistologic staining. Scale bars, 50 μm. **i** Relative mRNA expression of genes related to inflammation in liver. **j**-**k** Relative abundance of bacteria at the phylum (**j**) and genus (**k**) level in HFD and DOP groups. **l-n** α-diversity indexes in HFD and DOP group as indicated by the Shannon, ACE, and Chao1 indexes. The box plots showing the minima, maxima, centre, bounds of box and whiskers and percentile. **o** Principal coordinate analysis (PCoA) of the gut microbiota between the HFD and DOP groups by weighted UniFrac distance (ANOSIM test). **p** Taxonomic cladogram generated from LEfSe analysis. Each circle's size is proportional to the taxon's abundance. **q** Spearman correlations (two-tailed Spearman's rank test) between the top 20 genera and IR phenotypes. The color represents positive (red) or negative (blue) correlations and FDRs are denoted. *, FDR < 0.05; **, FDR < 0.01. DOP (*Dendrobium officinale* polysaccharide); HFD (High-fat diet). Data (a-g, i) are presented as the mean ± SEM. Statistical analysis was performed using One-way ANOVA with Tukey's post hoc test for (e), (f), and (i), One-way ANOVA with Dunnett's T3 post hoc test for (c) and (g), Kruskal-Wallis test for (a) and (b). Source data can be found in Source Data file.

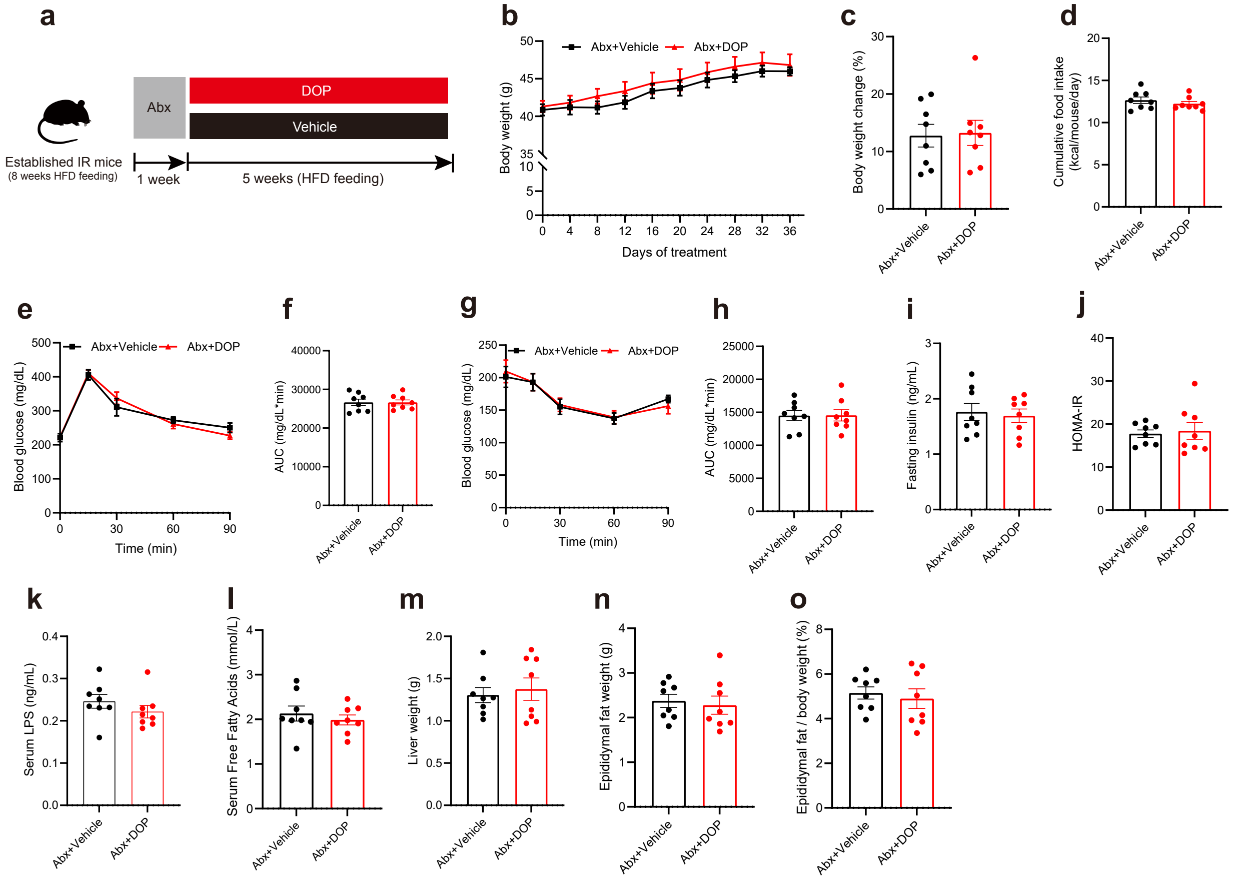

Supplementary Fig. 4 DOP ameliorates IR in a gut microbiota-dependent manner

Mice were treated with antibiotic cocktails (Abx) for 7 days to deplete gut microbiota, followed by DOP administration. n = 8 mice per group. **a** Experimental scheme for b to o. **b** Body weight curve. **c** Body weight change (%). **d** Food intake. **e-f** OGTT (**e**) and AUC (**f**). **g-h** ITT (**g**) and AUC (**h**). **i** Serum insulin. **j** HOMA-IR. **k** Serum LPS. **l** Serum free fatty acids. **m** Liver weight. **n** Epididymal fat weight. **o** Epididymal fat/body weight (%). DOP (*Dendrobium officinale* polysaccharide). Data are presented as the mean ± SEM. Source data can be found in Source Data file.

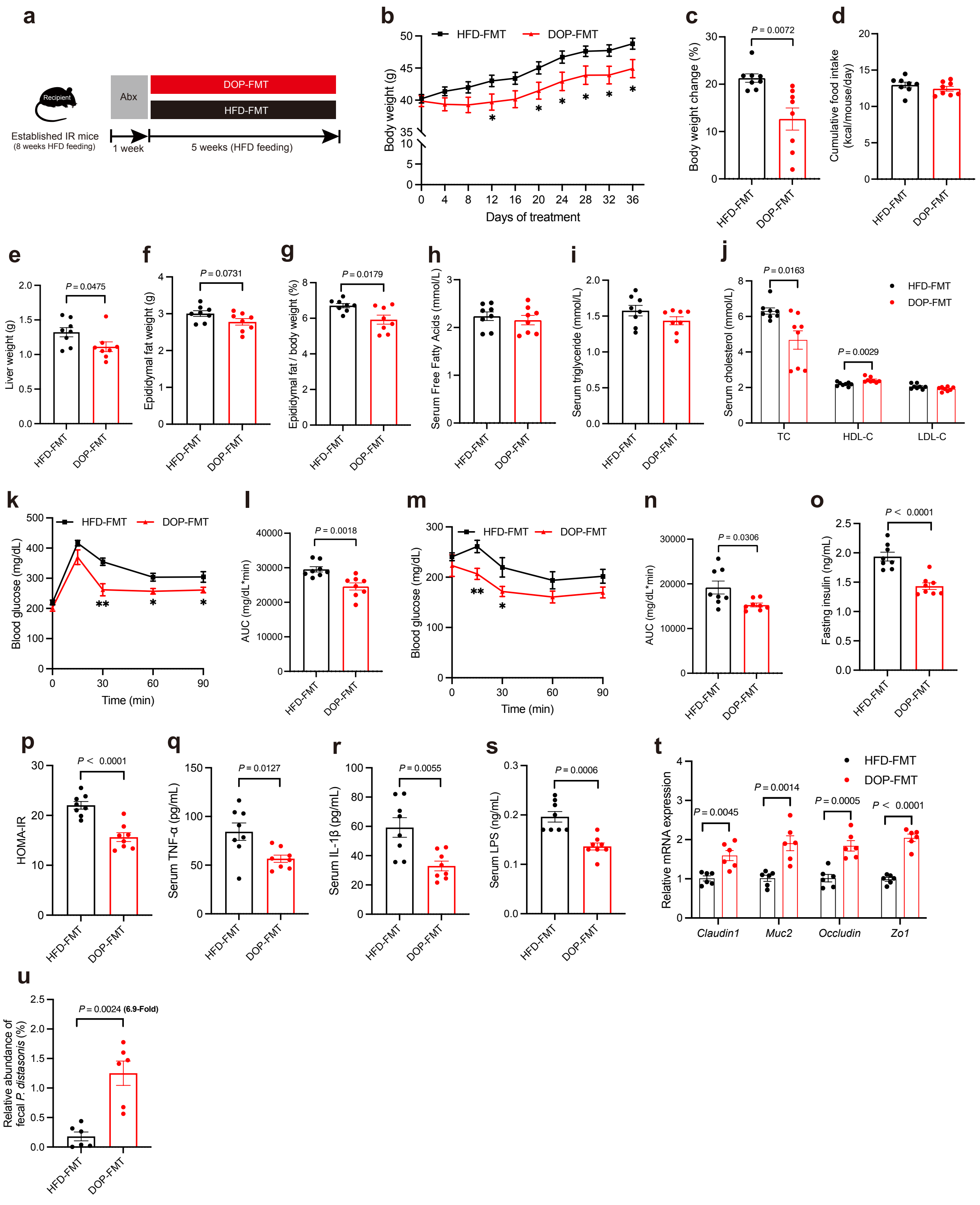

Supplementary Fig. 5 Fecal microbial transplantation of DOP-regulated microbiota mitigates HFD-induced IR

Fecal homogenates from DOP-treated or untreated mice were orally transmitted daily into antibiotic-treated recipient mice for 5 weeks. (b-s) n = 8 mice per group, (t-u) n = 6 mice per group. **a** Experimental scheme for b to u. **b** Body weight curve. **c** Body weight change (%). **d** Food intake. **e** Liver weight. **f** Epididymal fat weight. **g** Epididymal fat/body weight (%). **h** Serum free fatty acids. **i** Serum triglyceride. **j** Serum cholesterol. **k-l** OGTT (**k**) and AUC (**l**). **m-n** ITT (**m**) and AUC (**n**). **o** Serum insulin. **p** HOMA-IR. **q** Serum TNF-α. **r** Serum IL-1β. **s** Serum LPS. **t** Relative mRNA expression of genes related to intestinal permeability in colon. **u** Relative abundance of fecal *P. distasonis*. DOP (*Dendrobium officinale* polysaccharide); HFD (High-fat diet); FMT (fecal microbial transplantation). Data are presented as the mean ± SEM. Statistical analysis was performed using two-tailed unpaired t-test for (b), (d), (e), (f), (h), (i), HDL-c of (j), 30 min of (k), (l), (m), (n), (o), (p), (q), *Muc2*, *Occludin*, and *Zo1* of (t), two-tailed unpaired t-test with Welch's correction for (c), (g), TC of (j), (r), *Claudin1* of (t), and (u), two-tailed Mann-Whitney test for 60 and 90 min of (k) and (s). *, $P < 0.05$; **, $P < 0.01$. Source data can be found in Source Data file.

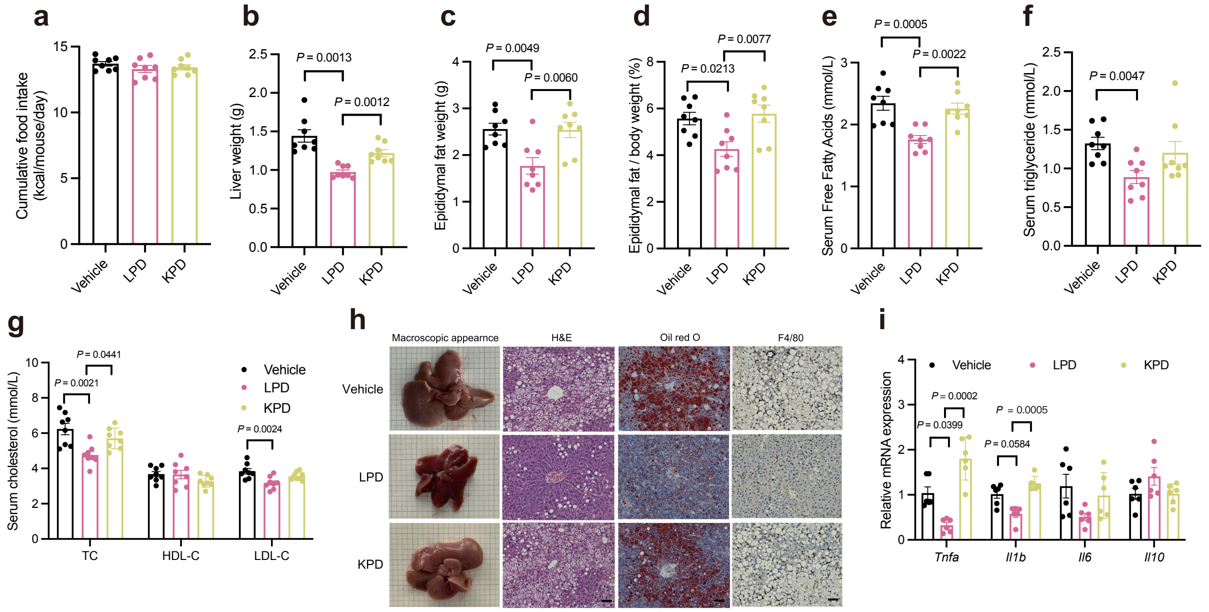

Supplementary Fig. 6 Effects of *P. distasonis* on HFD-induced IR

After an 8-week HFD treatment, mice were given PBS (Vehicle group), *P. distasonis* NSP007 (LPD group), or heat-killed *P. distasonis* NSP007 (KPD group) for 5 weeks. (a-g) n = 8 mice per group, (h-i) n = 6 mice per group. **a** Food intake. **b** Liver weight. **c** Epididymal fat weight. **d** Epididymal fat/body weight (%). **e** Serum free fatty acids. **f** Serum triglyceride. **g** Serum cholesterol. **h** Representative images of the appearance of liver, liver sections after H&E staining, oil red O staining, and F4/80 immunohistologic staining. Scale bars, 50 µm. **i** Relative mRNA expression of genes related to liver inflammation. Data are presented as the mean ± SEM. Statistical analysis was performed using One-way ANOVA with Tukey's post hoc test for (c), (d), (e), and LDL-c of (g), One-way ANOVA with Dunnett's T3 post hoc test for (b) and TC of (g), Kruskal-Wallis test for (f) and (i). Source data can be found in Source Data file.

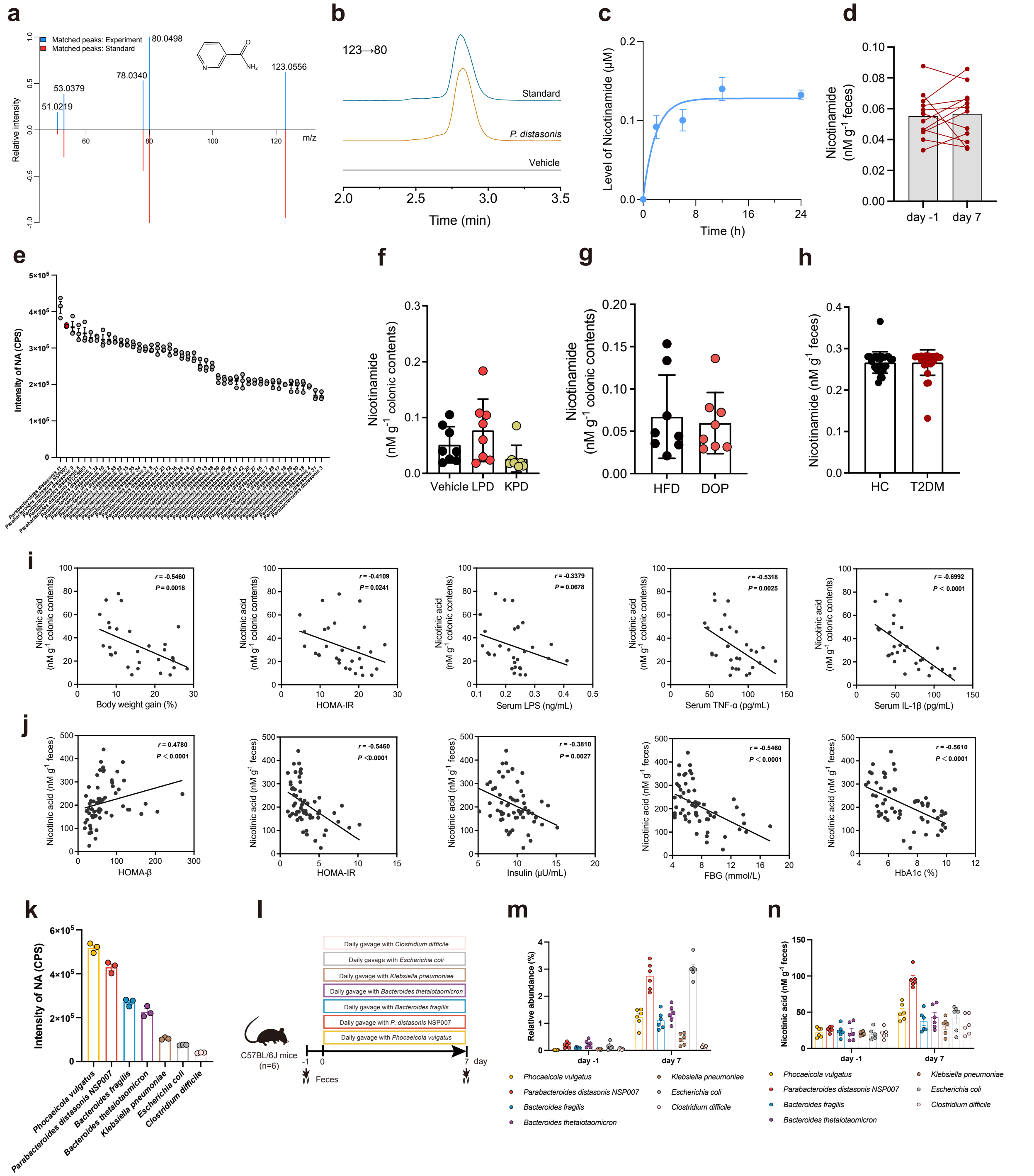

Supplementary Fig. 7 Nicotinic acid is a bioactive metabolite of *P. distasonis*

a The MS/MS spectra of NAM standard and NAM detected in *P. distasonis*-incubated samples. **b** Extracted ion chromatograms of NAM from cultured *P. distasonis* compared to the Vehicle. **c** The level of NAM in the culture supernatant of *P. distasonis* at different time points, n = 3 biologically independent samples per group. **d** The change of fecal NAM after gavage with *P. distasonis*. n = 12 mice per group. **e** The intensity

of NA in the culture supernatant of 44 *P. distasonis* after 24 h of incubation. n = 3 biologically independent samples per group. **f** Colonic NAM level in Vehicle, LPD, and KPD group of mice. n = 8 mice per group. **g** Colonic NAM level in HFD and DOP group of mice. n = 8 mice per group. **h** Fecal NAM level in HC (n = 30) and T2DM (n = 30) group of humans. **i** Spearman correlations (two-tailed Spearman's rank test) between the colonic NA level and the severity of IR in mice. **j** Spearman correlations (two-tailed Spearman's rank test) between the fecal NA level and the severity of IR in humans. **k** The intensity of NA in the culture supernatant of different strains after 24 h of incubation. n = 3 biologically independent samples per group. **l-n** The changes of fecal NA before and after colonization by different strains. Mice were treated with bacteria strains (2×10^8 CFU/mice/d) for 7 days. n = 6 mice per group. (**l**) Experimental scheme for m to n, (**m**) Relative abundance of different bacteria strains in feces before and after treatment. (**n**) The change of fecal NA level before and after different bacteria strains treatment. DOP (*Dendrobium officinale* polysaccharide); HFD (High-fat diet); LPD (*P. distasonis* NSP007); KPD (heat-killed *P. distasonis* NSP007); T2DM (type 2 diabetes mellitus); HC (health control). Data are presented as the mean ± SEM. Source data can be found in Source Data file.

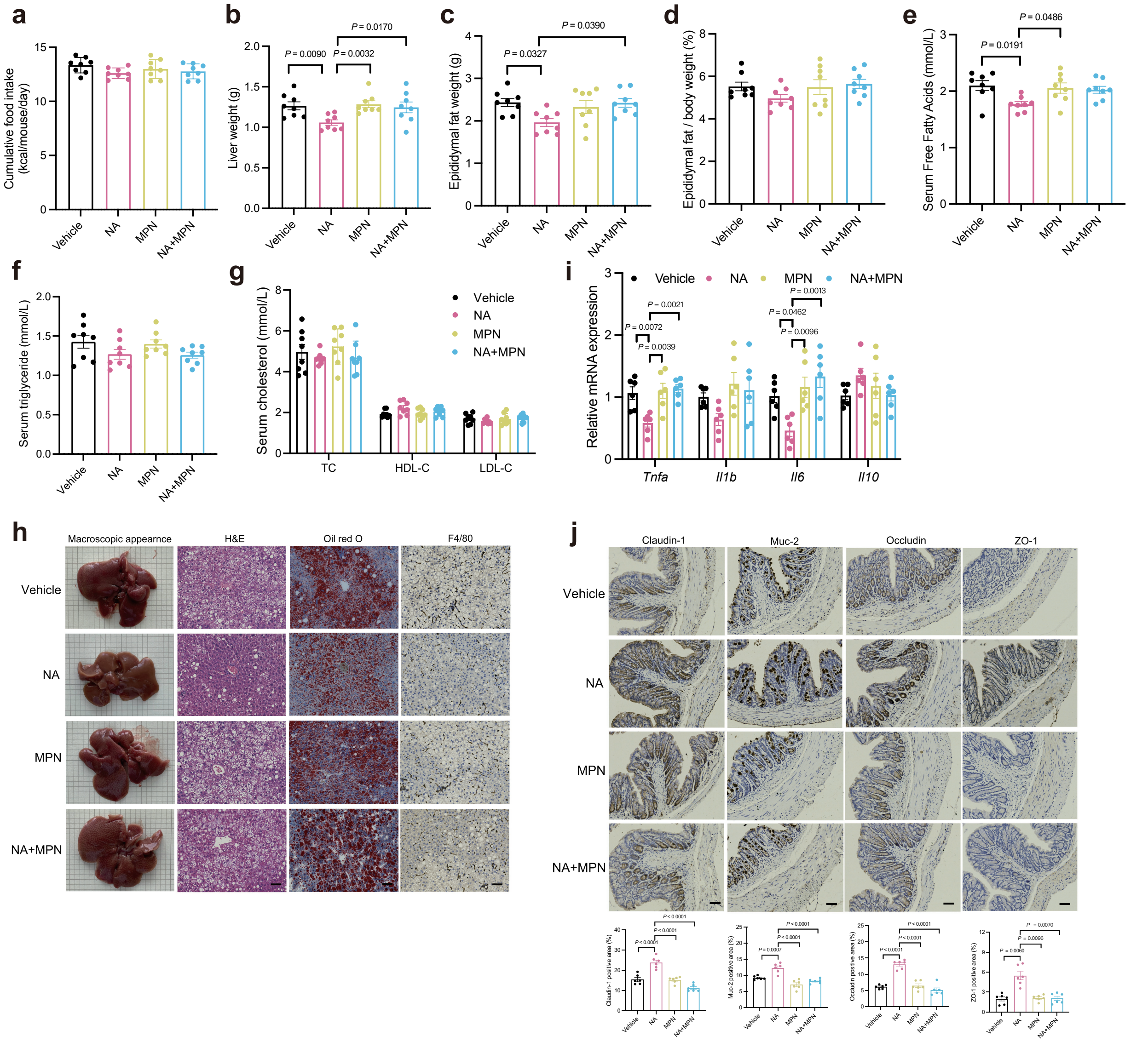

Supplementary Fig. 8 Nicotinic acid ameliorates HFD-induced IR

After an 8-week HFD treatment, mice were treated with PBS (Vehicle group), NA (NA group), MPN (Mepenzolate bromide, MPN group), or a combination of NA and MPN (NA+MPN group) three times a week for 5 weeks. (a-g) n = 8 mice per group, (i-j) n = 6 mice per group. **a** Food intake. **b** Liver weight. **c** Epididymal fat weight. **d** Epididymal fat/body weight (%). **e** Serum free fatty acids. **f** Serum triglyceride. **g** Serum cholesterol. **h** Representative images of the appearance of liver, liver sections after H&E staining, oil red O staining, and F4/80 immunohistologic staining. Scale bars, 50 μm. **i** Relative mRNA expression of genes related to liver inflammation. **j** Claudin-1, Muc-2, Occludin, and ZO-1 immunohistologic staining of colonic sections (top), and quantified positive area (bottom). Scale bars, 50 μm. Data are presented as the mean ± SEM. Statistical analysis was performed using One-way ANOVA with Tukey's post hoc test

for (c), (e), (i), and *Claudin1*, *Muc2*, and *Occludin* of (j), One-way ANOVA with Dunnett's T3 post hoc test for *Zo1* of (j), Kruskal-Wallis test for (b). Source data can be found in Source Data file.

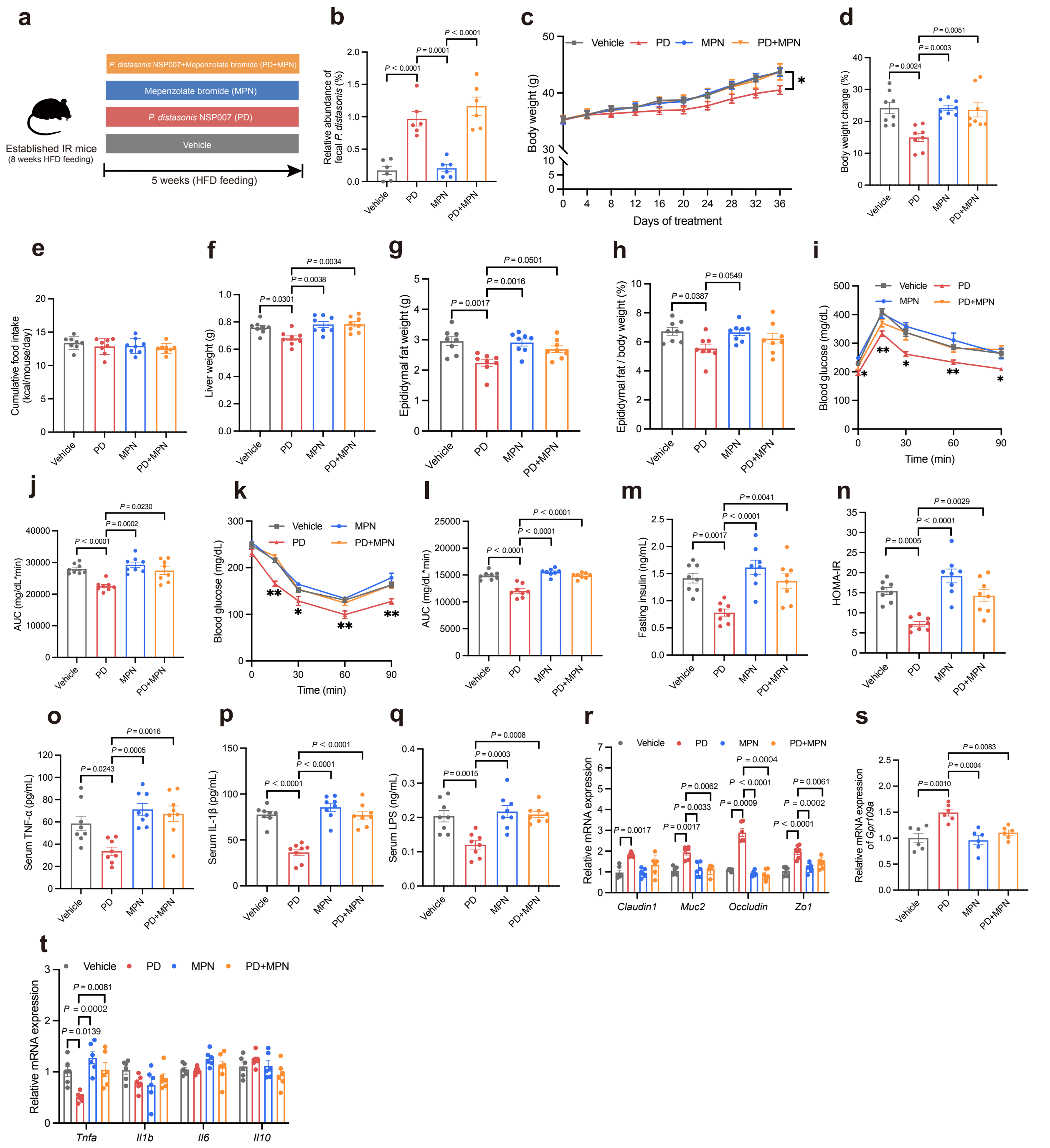

Supplementary Fig. 9 ***P. distasonis*** **ameliorates IR in a GPR109a activation-dependent manner**

Mice were fed an HFD for 8 weeks, then treated with PBS, *P. distasonis*, MPN, and *P. distasonis* + MPN for a further five weeks. (b-q) n = 8 mice per group, (r-t) n = 6 mice per group. **a** Experimental scheme for b to t. **b** Relative abundance of *P. distasonis* in feces accessed by qPCR. **c** Body weight curve. **d** Body weight change (%). **e** Food intake. **f** Liver weight. **g** Epididymal fat weight. **h** Epididymal fat/body weight

(%). **i-j** OGTT (**i**) and AUC (**j**). **k-l** ITT (**k**) and AUC (**l**). **m** Fasting insulin level. **n** HOMA-IR. **o** Serum TNF-α. **p** Serum IL-1β. **q** Serum LPS. **r** Relative mRNA expression of genes related to intestinal permeability in colon. **s** Relative mRNA expression of *Gpr109a* in colon. **t** Relative mRNA expression of genes related to inflammation in liver. Data are presented as the mean ± SEM. Statistical analysis was performed using One-way ANOVA with Tukey's post hoc test for (b), (f), (h), (i), (k), (l), (m), (n), (o), (p), (q), *Zo1* of (r), (s), and (t), One-way ANOVA with Dunnett's T3 post hoc test for (c), (j), *Claudin1*, and *Occludin* of (r), Kruskal-Wallis test for (d), (g) and *Muc2* of (r). *, $P < 0.05$; **, $P < 0.01$. Source data can be found in Source Data file.

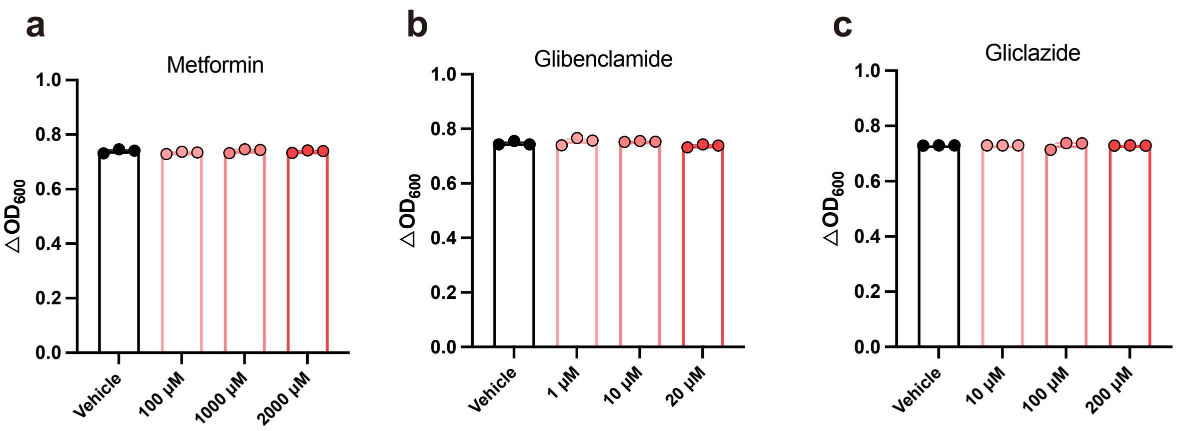

Supplementary Fig. 10 Effects of three main antidiabetic drugs taken by T2DM patients on the growth of *P. distasonis*.

a Metformin treatment. **b** Glibenclamide treatment. **c** Gliclazide treatment. Data are presented as the mean ± SEM. n = 3 biologically independent samples per group. Source data can be found in Source Data file.

Supplemental Tables

Supplementary Table. 1 Baseline characteristics of the study cohort

Characteristics	T2DM group (n = 30)	HC group (n = 30)	*P* Value
Age§, years	56.5 (52.5, 63)	54.5 (44.75, 61.5)	0.3037
Female†, %	20 (66.7)	24 (80.0)	0.2430
BMI§, kg/m^2	25.4 (23.9, 29.4)	25.6 (25.1, 26.3)	0.8130
SBP§, mmHg	126.5 (119, 151.5)	129.5 (124.5, 140.3)	0.7900
DBP*, mmHg	83.7 ± 12.1	78.8 ± 10.1	0.0939
HbA1c§, %	8.7 (7.9, 9.7)	5.7 (5.2, 6.1)	＜0.0001
FBG§, mmol/L	8.9 (7.3, 11.5)	5.7 (4.9, 6.2)	＜0.0001
Insulin*, µU/mL	10.6 ± 1.9	8.2 ± 1.8	＜0.0001
HOMA-IR§	4.0 (3.2, 5.4)	2.1 (1.6, 2.6)	＜0.0001
HOMA-β§	36.1 (29.1, 49.8)	87.1 (59.2, 117.1)	＜0.0001
TP*, g/L	78.0 ± 5.1	75.5 ± 5.3	0.0683
BUN§, mmol/L	5.4 (4.4, 6.0)	4.8 (3.8, 5.9)	0.2488
CREA§, µmol/L	47.2 (40.8, 55.5)	52.6 (45.3, 60.3)	0.0646
UA*, µmol/L	199.9 ± 59	225.3 ± 61.3	0.1071
CHOL*, mmol/L	4.9 ± 1.2	4.7 ± 1.1	0.4791
TG§, mmol/L	1.1 (0.6, 2.2)	1.0 (0.8, 0.7)	0.8476
HDL*, mmol/L	1.4 ± 0.3	1.5 ± 0.4	0.1238
LDL*, mmol/L	3.2 ± 1.0	2.9 ± 0.9	0.2543
ALT§, U/L	22.8 (19.6, 28.2)	23.1 (16.7, 29.1)	0.7618
AST§, U/L	21.1 (18.8, 23.8)	23.0 (20.0, 27.0)	0.0977
AST/ALT*	0.9 ± 0.3	1.1 ± 0.4	0.1196
Oral antidiabetic drugs, %	26 (86.7)	NA	
Metformin€, %	23 (76.7)	NA	
Glibenclamide€, %	7 (23.3)	NA	
Gliclazide€, %	7 (23.3)	NA	
Other drugs€, %	5 (16.7)	NA	
Current smoker†, %	3 (10.0)	2 (6.7)	0.6400
Drinking condition (Never/Sometimes/Often)	25/5/0	27/3/0	
Sweet food consumption (Never/Sometimes/Often)	23/1/6	17/5/8	

Statistical analysis was performed using two-tailed unpaired t-test for data marked with *, two-tailed Mann-Whitney test for data marked with §, and two-tailed Chi-Squared test for data marked with †. €Some patients take more than one antidiabetic drug.

Supplementary Table. 2 Result of spiked recoveries and relative standard deviations

Compound Name	Concentration (mg/g)	Spiked Concentration (mg/g)	Determined Concentration (mg/g)						Recovery (%)	RSD (%)
			1	2	3	4	5	6		
Nicotinic acid	3.152	1.00	4.055	4.097	4.114	4.167	4.324	4.274	100.629	3.34
		3.00	6.042	6.178	6.339	6.375	6.169	6.393	103.088	4.33
		6.00	8.975	9.043	9.056	9.146	9.177	9.032	97.446	2.46

Supplementary Table. 3 Baseline characteristics of validation cohort

Characteristics	T2DM group (n = 30)	HC group (n = 30)	P Value
Age*, years	55.1 ± 7.8	54.1 ± 8.7	0.6521
Female†, %	21 (70.0)	23 (76.7)	0.7703
BMI§, kg/m^2	25.1 (23.4, 29.1)	26.0 (25.3, 26.6)	0.5201
SBP*, mmHg	129.1 ± 7.9	128.5 ± 8.8	0.794
DBP§, mmHg	82.0 (79.8, 85.3)	82.5 (71.0, 87.0)	0.8070
HbA1c§, %	8.8 (8.1, 9.8)	5.4 (4.9, 5.8)	< 0.0001
FBG*, mmol/L	8.0 ± 0.7	5.3 ± 0.6	< 0.0001
Insulin#, µU/mL	9.8 ± 2.4	7.5 ± 1.2	< 0.0001
HOMA-IR§	3.6 (2.7, 4.1)	1.6 (1.5, 2.1)	< 0.0001
HOMA-β§	44.8 (33.5, 54.3)	88.8 (61.9, 106.5)	< 0.0001
TP*, g/L	72.4 (70.2, 75.9)	72.3 (70.0, 75.9)	0.5434
BUN#, mmol/L	4.8 ± 0.9	5.1 ± 1.5	0.3612
CREA§, µmol/L	50.4 (39.0, 61.6)	54.5 (48.3, 59.6)	0.1558
UA*, µmol/L	194.7 (139.8, 229.6)	217.2 (179.9, 252.9)	0.101
CHOL*, mmol/L	5.0 ± 1.1	4.9 ± 0.8	0.7262
TG§, mmol/L	1.4 (0.8, 1.9)	1.1 (0.7, 1.5)	0.1087
HDL#, mmol/L	1.4 ± 0.2	1.5 ± 0.5	0.1696
LDL#, mmol/L	3.0 (2.5, 3.6)	2.7 (2.4, 3.3)	0.0905
ALT#, U/L	24.9 (16.6, 32.2)	24.5 (19.1, 27.3)	0.352
AST*, U/L	23.2 (16.4, 25.4)	23.0 (19.0, 28.1)	0.1317
AST/ALT*	0.8 (0.7, 1.1)	1.0 (0.8, 1.2)	0.1736
Oral antidiabetic drugs, %	23 (76.7)	NA	
Metformin€, %	20 (66.7)	NA	
Glibenclamide€, %	8 (26.7)	NA	
Gliclazide€, %	3 (10.0)	NA	
Other drugs€, %	3 (10.0)	NA	
Current smoker†, %	2 (6.7)	2 (6.7)	1.0000
Drinking condition (Never/Sometimes/Often)	23/4/3	25/3/2	
Sweet food consumption (Never/Sometimes/Often)	27/1/2	25/3/2	

Statistical analysis was performed using two-tailed unpaired t-test for data marked with *, two-tailed unpaired t-test with Welch's correction for data marked with #, two-tailed Mann-Whitney test for data marked with §, and two-tailed Chi-Squared test for data marked with †. €Some patients take more than one antidiabetic drug.

Supplementary Table. 4 PCR primers used in this study

Mouse primers	Primer sequence (5'–3')	Bacterial primers	Primer sequence (5'–3')
Gpr109a[1]	GGGCTGGAATTTGTGTTCGGA	*P. distasonis*[2]	TGATCCCTTGTGCTGCT
	CTGTCGGTTCATAGCCAACAT		ATCCCCCTCATTCGGA
Zo1	CCGACGGCACGTCTCTC	*P. merdae*[3]	AGGGTGCGTAGGTGGTGAT
	GTCTCCTCCAAAGCCTGCTC		TTCACCGCTACACCACGC
Occludin	TTGAAAGTCCACCTCCTTACAGA	*P.goldsteinii*[4]	GCAGCACGATGTAGCAATACA
	CCGGATAAAAAGAGTACGCTGG		TTAACAAATATTTCCATGTGGAAC
Claudin1	CCTGCCCCAGTGGAAGATTT	*P. johnsonii*[5]	GCACAGATTCTACACTCCCCT
	AAACGCAGGACATCCACAGT		AGCAACGAAACAACCTGTGA
Muc2	ATGCCCACCTCCTCAAAGAC	*P. gordonii*[5]	TTCACCTTCTACGGCTACTACTACG
	GTAGTTTCCGTTGGAACAGTGAA		ACATAACGATCAAGGGTGCTGAAG
Tnfa	TAGCCAGGAGGGAGAACAGA	*F. prausnitzii*[6]	CCCGGCATCGGGTAGAG
	TTTTCTGGAGGGAGATGTGG		GGACGCGAGGCCATCTC
Il6	CCAAGAGGTGAGTGCTTCCC	*Bacteroides thetaiotaomicron*[7]	TACAATTGCCACAGTACGGAACA
	CTGTTGTTCAGACTCTCTCCCT		GCTGACGAACGATGACCATAGTTA
Il1b	TTGAAGAAGAGCCCATCCTC	*B. fragilis*[8]	TGATTCCGCATGGTTTCATT
	CAGCTCATATGGGTCCGAC		CGACCCATAGAGCCTTCATC
Il10	GCTCTTACTGACTGGCATGAG	*B. vulgatus*[9]	GCATCATGAGTCCGCATGTTC
	CGCAGCTCTAGGAGCATGTG		TCCATACCCGACTTTATTCCTT
β-actin	GGCTGTATTCCCCTCCATCG	*Clostridium difficile*[10]	TTGAGCGATTTACTTCGGTAAAGA
	CCAGTTGGTAACAATGCCATGT		CCATCCTGTACTGGCTCACCT
**Human primers**	**Primer sequence (5'–3')**	*Escherichia coli*[11]	CATGCCGCGTGTATGAAGAA
Gpr109a[12]	GCCCTTCCTGATGGACAAC		CGGGTAACGTCAATGAGCAAA
	TAGCCAACATGAAGAGCATC	*Klebsiella pneumoniae*[13]	ACGCTGTCACGACGCTATC
Zo1	ACCAGTAAGTCGTCCTGATCC		GGCTGAGGCACGGTATCC
	TCGGCCAAATCTTCTCACTCC	*Eubacteria* (all bacteria)[14]	ACTCCTACGGGAGGCAGCAG
Occludin	GACTTCAGGCAGCCTCGTTAC		ATTACCGCGGCTGCTGG
	GCCAGTTGTGTAGTCTGTCTCA		
Claudin1	CCTCCTGGGAGTGATAGCAAT		
	GGCAACTAAAATAGCCAGACCT		
Muc2	GAGGGCAGAACCCGAAACC		
	GGCGAAGTTGTAGTCGCAGAG		
β-actin	GCGGGAAATCGTGCGTGACATT		
	GATGGAGTTGAAGGTAGTTTCGTG		

References

1 Singh, N. et al. Activation of gpr109a, receptor for niacin and the commensal metabolite butyrate, suppresses colonic inflammation and carcinogenesis. *Immunity* **40**, 128-139, (2014).

2 Liu, C. X. et al. Rapid identification of the species of the Bacteroides fragilis group by multiplex PCR assays using group- and species-specific primers. *FEMS Microbiol. Lett.* **222**, 9-16, (2003).

3 Tong, J., Liu, C., Summanen, P., Xu, H. & Finegold, S. M. Application of quantitative real-time PCR for rapid identification of Bacteroides fragilis group and related organisms in human wound samples. *Anaerobe* **17**, 64-68, (2011).

4 Ishibashi, R. et al. Isoliquiritigenin attenuates adipose tissue inflammation and metabolic syndrome by modifying gut bacteria composition in mice. *Mol. Nutr. Food Res.* **66**, e2101119, (2022).

5 Tanoue, T. et al. A defined commensal consortium elicits CD8 T cells and anti-cancer immunity. *Nature* **565**, 600-605, (2019).

6 Machiels, K. et al. A decrease of the butyrate-producing species Roseburia hominis and Faecalibacterium prausnitzii defines dysbiosis in patients with ulcerative colitis. *Gut* **63**, 1275-1283, (2014).

7 Nakanishi, H. et al. Identification of feces by detection of Bacteroides genes. *Forensic Science International-Genetics* **7**, 176-179, (2013).

8 Zeng, S.-L. et al. Citrus polymethoxyflavones attenuate metabolic syndrome by regulating gut microbiome and amino acid metabolism. *Science Advances* **6**, eaax6208, (2020).

9 Ishaq, H. M. et al. Molecular alteration analysis of human gut microbial composition in Graves' disease patients. *International Journal of Biological Sciences* **14**, 1558-1570, (2018).

10 Collins, J. et al. Dietary trehalose enhances virulence of epidemic Clostridium difficile. *Nature* **553**, 291-294, (2018).

11 Huijsdens, X. W. et al. Quantification of bacteria adherent to gastrointestinal mucosa by real-time PCR. *Journal of Clinical Microbiology* **40**, 4423-4427, (2002).

12 Maciejewski-Lenoir, D. et al. Langerhans cells release prostaglandin D-2 in response to nicotinic acid. *Journal of Investigative Dermatology* **126**, 2637-2646, (2006).

13 Xie, S.-T. et al. VFG-Chip: A high-throughput qPCR microarray for profiling virulence factor genes from the environment. *Environment International* **172**, 107761, (2023).

14 Haarman, M. & Knol, J. Quantitative real-time PCR analysis of fecal Lactobacillus species in infants receiving a prebiotic infant formula. *Applied and Environmental Microbiology* **72**, 2359-2365, (2006).
