# Supplementary material for: Parabacteroides distasonis ameliorates insulin resistance via activation of intestinal GPR109a
Source: Nat Commun. 2023 Nov 25;14:7740. doi: 10.1038/s41467-023-43622-3 (PMC10676405; doi:10.1038/s41467-023-43622-3)
Supplement: Supplementary file 3 — Reporting Summary [file 41467_2023_43622_MOESM3_ESM.pdf]

nature portfolio

Corresponding author(s): Shaoping Nie
Last updated by author(s): Nov 7, 2023

Reporting Summary

Nature Portfolio wishes to improve the reproducibility of the work that we publish. This form provides structure for consistency and transparency in reporting. For further information on Nature Portfolio policies, see our Editorial Policies and the Editorial Policy Checklist.

Statistics

For all statistical analyses, confirm that the following items are present in the figure legend, table legend, main text, or Methods section.

n/a	Confirmed	
☐	☒	The exact sample size (n) for each experimental group/condition, given as a discrete number and unit of measurement
☐	☒	A statement on whether measurements were taken from distinct samples or whether the same sample was measured repeatedly
☐	☒	The statistical test(s) used AND whether they are one- or two-sided<br>*Only common tests should be described solely by name; describe more complex techniques in the Methods section.*
☒	☐	A description of all covariates tested
☐	☒	A description of any assumptions or corrections, such as tests of normality and adjustment for multiple comparisons
☐	☒	A full description of the statistical parameters including central tendency (e.g. means) or other basic estimates (e.g. regression coefficient) AND variation (e.g. standard deviation) or associated estimates of uncertainty (e.g. confidence intervals)
☐	☒	For null hypothesis testing, the test statistic (e.g. F, t, r) with confidence intervals, effect sizes, degrees of freedom and P value noted<br>*Give P values as exact values whenever suitable.*
☒	☐	For Bayesian analysis, information on the choice of priors and Markov chain Monte Carlo settings
☒	☐	For hierarchical and complex designs, identification of the appropriate level for tests and full reporting of outcomes
☐	☒	Estimates of effect sizes (e.g. Cohen's d, Pearson's r), indicating how they were calculated

Our web collection on statistics for biologists contains articles on many of the points above.

Software and code

Policy information about availability of computer code

Data collection	16S rRNA gene sequencing were collected by Illumina Miseq platform. Metabolite content were collected by TripleTOF 5600 or QTRAP 4500 system. Other data were collected with Microsoft Excel 2019.
Data analysis	All charts were generated using GraphPad Prism (version, 9.0). SPSS (version 26.0) was used for statistical analysis. The volcano map was performed using Bioinformatics tools. The metabolites were quantified using Progenesis QI software (version, 2.0) and MultiQuant software (version 3.0.1).

For manuscripts utilizing custom algorithms or software that are central to the research but not yet described in published literature, software must be made available to editors and reviewers. We strongly encourage code deposition in a community repository (e.g. GitHub). See the Nature Portfolio guidelines for submitting code & software for further information.

Data

Policy information about availability of data

All manuscripts must include a data availability statement. This statement should provide the following information, where applicable:

- Accession codes, unique identifiers, or web links for publicly available datasets
- A description of any restrictions on data availability
- For clinical datasets or third party data, please ensure that the statement adheres to our policy

The 16S rRNA data generated in this study were deposited in the NCBI SRA database (https://www.ncbi.nlm.nih.gov/sra, accession code PRJNA1030208). The mass spectrometry data generated in this study were deposited in the MetaboLights under accession code MTBLS7943. All other data generated or analyzed during this

study are included in this published article (and its supplementary information files). Source data are provided with this paper.

Research involving human participants, their data, or biological material

Policy information about studies with human participants or human data. See also policy information about sex, gender (identity/presentation), and sexual orientation and race, ethnicity and racism.

Reporting on sex and gender	This study focused on the relationship between gut microbiota and insulin resistance. We included 60 type 2 diabetes mellitus (T2DM) patients (for cohort 1, 10 males and 20 females; for cohort 2, 9 males and 21 females) and 60 healthy adults (for cohort 1, 6 males and 24 females; for cohort 2, 7 males and 23 females) for analysis. The information regarding the sex of each participant was collected by self-reporting via a questionnaire.
Reporting on race, ethnicity, or other socially relevant groupings	All participants were Asian. The information regarding the race of each participant was collected by self-reporting via a questionnaire.
Population characteristics	A case-control study was proposed in which 60 type 2 diabetes mellitus patients (For cohort 1, n = 30; For cohort 2, n = 30) aged 25-70 years with abnormal blood glucose levels were selected and matched 1:1 to a healthy control population according to age, sex, and BMI, respectively.
Recruitment	All participants were recruited through notices in the First Affiliated Hospital of Nanchang University and Nanchang University. All of the participants provided written informed consent. There was no self-selection or artificial exclusion as volunteers were enrolled consecutively.
Ethics oversight	The study was approved by the Ethics Committee of the First Affiliated Hospital of Nanchang University, number IIT2022076. The trial was registered at the Chinese clinical trial registry, number ChiCTR2200065715.

Note that full information on the approval of the study protocol must also be provided in the manuscript.

Field-specific reporting

Please select the one below that is the best fit for your research. If you are not sure, read the appropriate sections before making your selection.

☒ Life sciences ☐ Behavioural & social sciences ☐ Ecological, evolutionary & environmental sciences

For a reference copy of the document with all sections, see nature.com/documents/nr-reporting-summary-flat.pdf

Life sciences study design

All studies must disclose on these points even when the disclosure is negative.

Sample size	This study had at least 3 samples or replicates for each experiment. For animal experiments, there were 8 mice per group in order to obtain statistical significance, which was similar to those previously used by us and others in this field (ref. 8, 10, and Nature. 2022; 610(7932): 562-568).
Data exclusions	No data was excluded.
Replication	All data is representative of two or more independent experiments and got similar results.
Randomization	High-fat-diet (HFD)-induced insulin resistance mice were randomly divided into experimental groups, with 8 mice per group, and the groups did not present differences in body weights before the treatments. For sample testing (such as mass spectrometry analysis), samples were processed and tested randomly.
Blinding	Investigators were blinded to group allocation during data collection and analysis. During the treatment of live animals, cages during sample collection and processing were labeled as code names that were later revealed by the individuals who picked and treated animals, but did not participate in sample collection and processing.

Reporting for specific materials, systems and methods

We require information from authors about some types of materials, experimental systems and methods used in many studies. Here, indicate whether each material, system or method listed is relevant to your study. If you are not sure if a list item applies to your research, read the appropriate section before selecting a response.

Materials & experimental systems

n/a	Involved in the study	
☐	☒	Antibodies
☐	☒	Eukaryotic cell lines
☒	☐	Palaeontology and archaeology
☐	☒	Animals and other organisms
☐	☒	Clinical data
☒	☐	Dual use research of concern
☒	☐	Plants

Methods

n/a	Involved in the study	
☒	☐	ChIP-seq
☒	☐	Flow cytometry
☒	☐	MRI-based neuroimaging

Antibodies

Antibodies used	Anti-Muc2 (Abcam, cat. # ab272692, 1:2000 for IHC),<br>Anti-F4/80 (Cell Signaling Technology, cat. # 70076S, 1:500 for IHC),<br>Anti-ZO1 (Abcam, cat. # ab221547, 1:500 for IHC),<br>Anti-Occludin (Cell Signaling Technology, cat. # 91131, 1:400 for IHC),<br>Anti-Claudin-1 (Abcam, cat. # ab307692, 1:100 for IHC).
Validation	1. Anti-Muc2 (Abcam, cat. # ab272692)<br>https://www.abcam.com/products/primary-antibodies/muc2-antibody-epr23479-47-ab272692.html<br>The manufacturer has validated this antibody for IHC in the species mouse.<br>2. Anti-F4/80 (Cell Signaling Technology, cat. # 70076)<br>https://www.cellsignal.com/product/productDetail.jsp?productId=70076<br>The manufacturer has validated this antibody for IHC in the species mouse.<br>3. Anti-ZO1 (Abcam, cat. # ab221547)<br>https://www.abcam.com/products/primary-antibodies/zo1-tight-junction-protein-antibody-epr19945-296-ab221547.html<br>The manufacturer has validated this antibody for IHC in the species mouse.<br>4. Anti-Occludin (Cell Signaling Technology, cat. # 91131)<br>https://www.cellsignal.com/products/primary-antibodies/occludin-e6b4r-rabbit-mab/91131<br>The manufacturer has validated this antibody for IHC in the species mouse.<br>5. Anti-Claudin-1 (Abcam, cat. # ab307692).<br>https://www.abcam.com/products/primary-antibodies/claudin-1-antibody-epr25359-48-ab307692.html<br>The manufacturer has validated this antibody for IHC in the species mouse.

Eukaryotic cell lines

Policy information about cell lines and Sex and Gender in Research

Cell line source(s)	Human Caco-2 cells (ATCC, Cat#HTB-37, isolated from colon tissue of a 72-year-old white male person)
Authentication	The cell line used was authenticated by STR profiling
Mycoplasma contamination	The cell line was tested negative for mycoplasma contamination.
Commonly misidentified lines (See ICLAC register)	No misidentified line was used in the study

Animals and other research organisms

Policy information about studies involving animals; ARRIVE guidelines recommended for reporting animal research, and Sex and Gender in Research

Laboratory animals	5 weeks old C57BL/6J mice were purchased from Charles River Laboratories (Beijing, China). All mice were housed under specific pathogen-free conditions with a 12-h light-dark cycle and provided with standard chow and water ad libitum, with temperature kept at 21-24 °C, and humidity at 40-70%.
Wild animals	This study did not involve wild animals.
Reporting on sex	Findings from our study apply to male mice. The reason we selected male C57BL/6J mice is that male mice are more susceptible than female mice (PMID: 33692086; PMID: 19088436), which enhances the success rate of modeling.
Field-collected samples	Field-collected samples were not used in this study.

Ethics oversight	All animal experiments were performed under the Guidelines for Care and Use of Laboratory Animals of the National Institutes of Health and were approved by the Experimental Animal Care and Use Committee of Nanchang University, number IACUC-20221030002.

Note that full information on the approval of the study protocol must also be provided in the manuscript.

Clinical data

Policy information about clinical studies

All manuscripts should comply with the ICMJEguidelines for publication of clinical research and a completedCONSORT checklist must be included with all submissions.

Clinical trial registration	ChiCTR2200065715
Study protocol	The full trial protocol can be accessed in Supplementary information 2_Clinical trial protocol.docx
Data collection	The period for recruitment and data collection was 2022.11.10 -2022.12.15 and 2022.12.15-2022.12.31, respectively. The data were collected at the First Affiliated Hospital of Nanchang University.
Outcomes	Fecal microbiota was detected using 16S rRNA gene sequencing technology or qPCR quantitative technique. Fecal metabolites (especially NA) were detected using LC-MS. Serum biochemistry tests were detected by Automated Chemistry Analyzer.
